# Meta-Analysis of Associations of IL1 Receptor Antagonist and Estrogen Receptor Gene Polymorphisms with Systemic Lupus Erythematosus Susceptibility

**DOI:** 10.1371/journal.pone.0109712

**Published:** 2014-10-06

**Authors:** Li Cai, Jin-wei Zhang, Xing-xin Xue, Zhi-gang Wang, Jia-jia Wang, Shai-di Tang, Shao-wen Tang, Jie Wang, Yun Zhang, Xian Xia

**Affiliations:** 1 Department of Epidemiology and Biostatistics, School of Public Health, Nanjing Medical University, Jiangning District, Nanjing, Jiangsu, China; 2 Department of Anesthesiology, Affiliated Drum Tower Hospital of Medical College of Nanjing University, Gulou District, Nanjing, Jiangsu, China; 3 Department of Nosocomial Infection Control, General Hospital of Beijing Military Region, Dongcheng District, Beijing, China; 4 State Key Laboratory of Reproductive Medicine, Nanjing Medical University, Jiangning District, Nanjing, Jiangsu, China; 5 Department of General Practice, Kangda College, Nanjing Medical University, Jiangning District, Nanjing, Jiangsu, China; 6 Institute of Epidemiology and Microbiology, Huadong Research Institute for Medicine and Biotechnics, Nanjing, Jiangsu, China; University of Texas Health Science Center at Houston, United States of America

## Abstract

Systemic lupus erythematosus (SLE) is an autoimmune disease that affects a number of different organs and tissues. Interleukin-1 (IL1) and estrogen are considered potential elements in the pathology of SLE. Recently, the variable number of tandem repeats (VNTR) polymorphism in the IL1 receptor antagonist gene (*IL1-RN*) and *Pvu*II (rs2234693) and *Xba*I (rs9340799) polymorphisms in the estrogen receptor 1 gene (*ESR1*) have been associated with a predisposition to SLE. However, the evidence for these associations is inconclusive. We therefore conducted a meta-analysis to validate the roles of these polymorphisms in SLE susceptibility. We searched four databases and identified a total of 17 eligible articles comprising 24 studies. The Newcastle-Ottawa quality assessment scale was used to assess the qualities of the selected studies. We assessed the strengths of the associations using odds ratios (ORs) with 95% confidence intervals (95% CIs). Regarding the *IL-1RN* VNTR, the 2 allele significantly increased SLE susceptibility (2 vs. L: OR = 1.34, 95% CI = 1.03–1.73, *P* = 0.03). The *ESR1 Pvu*II CC/CT genotype was also associated with SLE susceptibility (CC/CT vs. TT: OR = 1.25, 95% CI = 1.06–1.47, *P* = 0.01), and the difference was especially pronounced among Asians (CC/CT vs. TT: OR = 1.33, 95% CI = 1.04–1.69, *P* = 0.02). No significant association between the *ESR1 Xba*I polymorphism and SLE susceptibility was observed in the overall analysis. However, a marginally significant association between the GG/GA genotype was found in individuals of Asian descent (GG/GA vs. AA: OR = 1.30, 95% CI = 1.01–1.67, *P* = 0.04). These results indicate that the *IL1-RN* VNTR 2 allele, *ESR1 Pvu*II CC/CT genotype and *ESR1 Xba*I GG/GA genotype may increase SLE susceptibility, especially in Asian individuals.

## Introduction

Systemic lupus erythematosus (SLE) is an autoimmune disease that affects various organs and tissues, involving the production of a range of autoantibodies against serological, intracellular, nucleic acid and cell surface antigens [1]. Although the mechanisms underlying SLE are not fully understood, genetic, environmental and hormonal factors are all thought to impact on the development of the disease [2].

Cytokines are considered to be potential elements in the pathology of SLE. These include interleukin-1 (IL1), which plays a key regulatory role in initiating and modulating immunologic and inflammatory events [3], [4]. Animals with experimental SLE produced increased levels of IL1 throughout the disease course [5]. The IL1 family consists of IL1α, IL1β and IL1 receptor antagonist (IL1-RA) [6]. IL1-RA is an important anti-inflammatory molecule that binds to IL1 receptors in competition with IL1α and IL1β, thus inhibiting their activities and modulating a variety of IL1-related immune and inflammatory activities [7]. The IL1-RA gene (*IL1-RN*) has a variable number of tandem repeats (VNTR) polymorphism of 86 base pairs (bp) in intron 2. Five alleles correspond to allele 1 (four repeats), allele 2 (two repeats), allele 3 (five repeats), allele 4 (three repeats) and allele 5 (six repeats), which can be further categorized into a long allele (L: 3–6 repeats) and a short allele (2∶2 repeats). The genotypes are therefore classified as LL, 2L and 22 [8]. Blakemore *et al*. [9] first revealed that the frequency of the *IL1-RN* VNTR 2 allele was increased in SLE patients, since when mounting studies have explored the relationship between the *IL1-RN* VNTR polymorphism and SLE susceptibility in different populations; however, the findings have been controversial [10]–[12].

Estrogen is another underlying element in the pathology of SLE. SLE typically presents in women of childbearing age [13] and its morbidity falls remarkably after the menopause, in line with the decline in endogenous estrogen [14]. In an SLE mouse model, female mice had poorer outcomes than male mice, and estrogens exacerbated, while androgens ameliorated, the disease [15]. One possible mechanism is that physiological concentrations of estrogen could affect the secretion of cytokines such as IL1 [16]–[18]. However, the roles of estrogen and IL1 in SLE remain unclear. Estrogen acts on target cells through binding to estrogen receptors (ERs). ERα, encoded by the ER 1 gene (*ESR1*), is the main form of ER. Two polymorphisms, *ESR1 Pvu*II T/C (rs2234693) and *ESR1 Xba*I A/G (rs9340799), located in the first intron of the *ESR1* gene, have been extensively studied, but the associations between these polymorphisms and SLE susceptibility remain controversial [19], [20].

Limited sample sizes and inadequate statistical power mean that the results of studies of the relationships between the *IL1-RN* VNTR, *ESR1 Pvu*II, and *ESR1 Xba*I polymorphisms and SLE susceptibility remain conflicting, rather than conclusive [9]–[12], [19]–[31]. Given the potentially important roles of these three polymorphisms in the pathological process and the increasing numbers of studies in different populations, we performed a meta-analysis to derive a more precise estimation of the associations between the *IL1-RN* VNTR, *ESR1 Pvu*II, and *ESR1 Xba*I polymorphisms and SLE susceptibility.

## Materials and Methods

### Search strategy

We searched the PubMed, Embase, Wanfang and Chinese National Knowledge Infrastructure databases using the search terms: ‘systemic lupus erythematosus’ or ‘SLE’, ‘polymorphism’ or ‘allele’ or ‘genotype’, ‘interleukin-1 receptor antagonist’ or ‘*IL1-RN*’ or ‘estrogen receptor’ or ‘*ER*’. The literature search was updated on December 2013 and there was no date limit. The results were also supplemented with manual searches of references from the final published articles.

### Study selection

The inclusion criteria were: (1) case-control design; (2) studies investigating the relationship between the *IL1-RN* VNTR, *ESR1 Pvu*II or *ESR1 Xba*I polymorphisms and SLE susceptibility; (3) studies with sufficient data to provide odds ratios (ORs) and 95% confidence intervals (CIs); and (4) diagnosis of SLE patients performed according to the American College of Rheumatology criteria [32], [33]. The exclusion criteria were: (1) studies with overlapping populations; and (2) studies with insufficient data.

### Data extraction

The following information was sought from each publication: first author’s surname, year of publication, participants’ country, ethnicity, sex distribution, genotyping methods, the source of control groups (population-based or hospital-based controls) and matching numbers of genotyped cases and controls. The literature search, eligible study selection and data extraction were carefully conducted independently by two reviewers (Cai and Zhang) and consensuses were reached on all items.

### Quality appraisal

Two reviewers (Cai and Zhang) independently rated the methodological quality of every included study according to the Newcastle-Ottawa quality assessment scale [34]. This scale contains nine items (1 point for each) in three parts: selection (four items), comparability (two items) and exposure (three items).

### Statistical analysis

Statistical manipulations were conducted using Stata 10.0 (Stata Corporation, College Station, TX, USA). A χ^2^ test for goodness of fit was used to test for Hardy-Weinberg equilibrium (HWE) in the control group, with *P*<0.05 indicating a deviation from HWE. Crude ORs and 95% CIs were used to assess the strengths of the associations between the *IL1-RN* and *ESR1* polymorphisms and SLE susceptibility. The statistical significance of the pooled ORs was determined by the Z test, with *P*<0.05 considered significant. Statistical heterogeneity among studies was assessed by the Q-test and the I^2^ statistic was used to estimate heterogeneity quantitatively [35]. In the absence of heterogeneity (*P*>0.10 or I^2^<50%), pooled ORs and 95% CIs were calculated by the fixed-effects model (Mantel-Haenszel method), otherwise the random-effects model (DerSimonian-Laird method) was used. Sensitivity analysis was used to evaluate the stability of the results of the meta-analysis by removing one study at a time to determine the influence of the individual data set on the pooled OR. Potential publication bias was examined by a funnel plot of log OR against its standard error using Begg’s test, and the degree of asymmetry was assessed by Egger’s unweighted regression asymmetry test. Publication bias may be present if *P*<0.05 [36].

## Results

### Study characteristics

**Figure 1:**
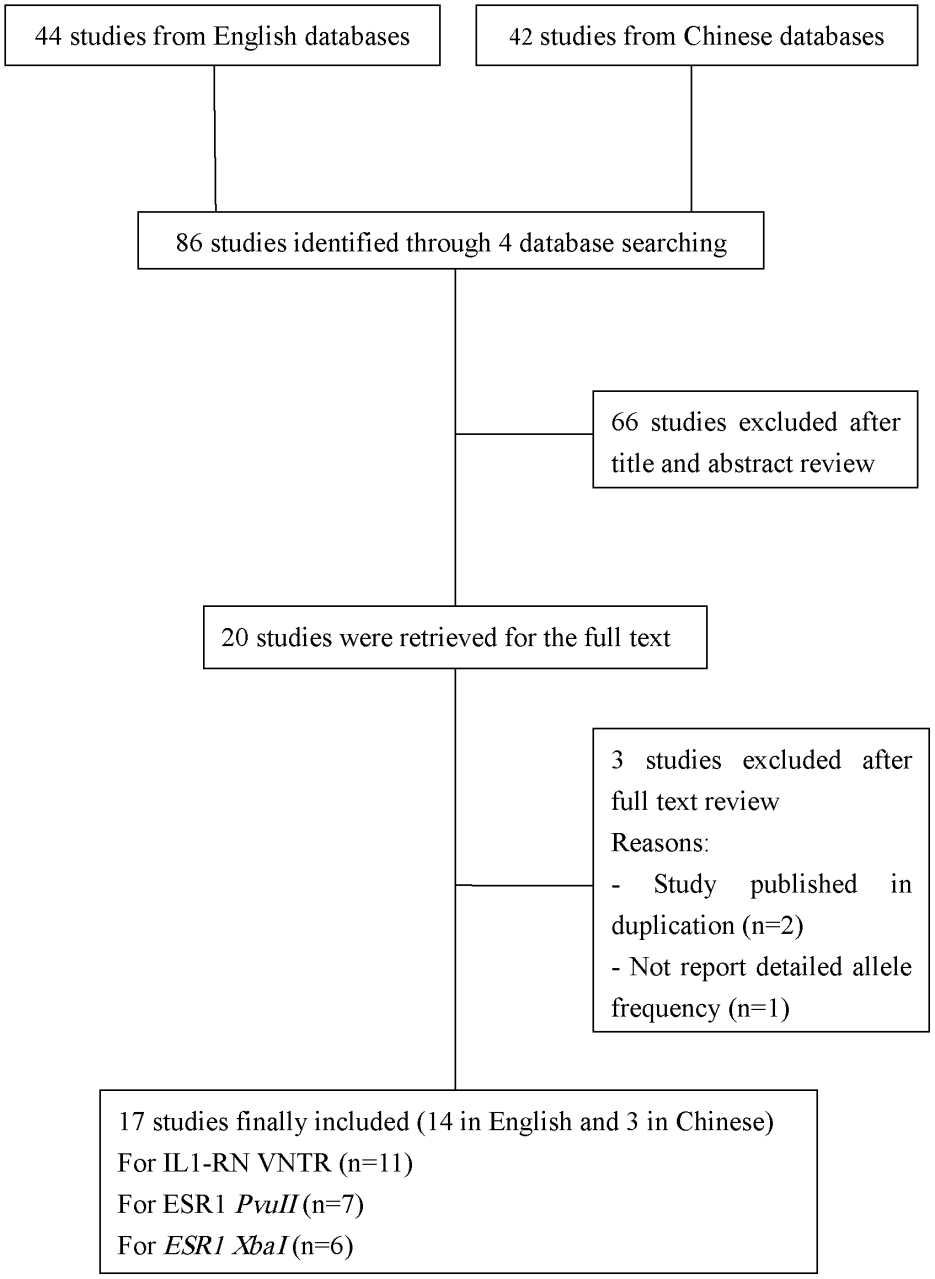
Flow diagram of studies included in the meta-analysis.

A detailed flow chart of the inclusion and exclusion processes is presented in Figure 1. Overall, 86 studies (44 in English and 42 in Chinese) were retrieved based on the search terms. Of these, 69 studies were excluded according to the inclusion and exclusion criteria and 17 eligible articles representing 24 studies were identified (one article was considered as several separate studies if it involved different populations or different target single nucleotide polymorphisms). Eleven studies including 1171 SLE patients and 1834 controls described *IL-1RN* VNTR genotypes, seven studies including 1012 SLE patients and 2442 controls described *ESR1 Pvu*II genotypes and six studies including 816 SLE patients and 1478 controls described *ESR1 Xba*I genotypes. The first author’s surname, publication year, ethnicity, quality score, sex distribution, frequencies of various genotypes in SLE patients and controls and HWE in controls for each study are listed in Tables 1, 2 and 3. The mean score of the quality appraisal was 6.54. In addition, most of the eligible studies were population-based and polymerase chain reaction was performed in all studies. Genotype distributions in the control populations agreed with HWE in all except seven studies [12], [19], [22], [23], [25], [29], [30].

**Table 1:** Characteristics and *IL1-RN* VNTR polymorphism genotype distributions in studies included in the meta-analysis.

Author, year	Ethnicity	Quality score^a^	Control					Case					*P*_HWE_
			LL	2L	22	L	2	LL	2L	22	L	2	
Tsai 2006 [10]	Taiwan(Asian)	6	–	–	–	142	6	–	–	–	198	10	–
Lee 2004 [11]	Korean(Asian)	7	109	18	0	236	18	83	10	0	176	10	0.39
Parks 2004 [12]	United States(Caucasian)	7	169	18	15	356	48	66	12	8	144	28	**<0.01**
Parks 2004 [12]	United States(African-American)	7	69	3	0	141	3	137	6	1	280	8	0. 86
Jonsen2004 [21]	Sweden(Caucasian)	6	111	75	14	297	103	86	38	14	210	66	0.78
Huang 2002 [22]	Taiwan(Asian)	7	96	6	1	198	8	43	8	1	94	10	**0.03**
Zhu 2000 [23]	China(Asian)	5	15	31	4	61	39	26	52	2	104	56	**0.03**
Tjernstrom 1999 [24]	Sweden(Caucasian)	7	–	–	–	339	39	–	–	–	130	32	–
Heward 1999 [25]	Caucasian(Caucasian)	4	312	7	19	631	45	106	4	6	216	16	**<0.01**
Suzuki 1997 [26]	Japan(Asian)	4	–	–	–	418	18	–	–	–	354	38	–
Blakemore 1994 [9]	England(Caucasian)	7	152	92	17	396	126	39	31	11	109	53	0.54

a The quality score was determinded by using the Newcastle-Ottawa quality assessment scale.

*IL1-RN*: Interleukin-1 receptor antagonist gene; VNTR: variable number of tandem repeats; HWE: Hardy-Weinberg equilibrium.

**Table 2:** Characteristics and *ESR1 Pvu*II polymorphism genotype distributions in studies included in the meta-analysis.

Author, year	Ethnicity	Quality score^a^	Gender (M/F)		Control					Case					*P*_HWE_
			Control	Case	TT	TC	CC	T	C	TT	TC	CC	T	C	
Kisiel 2011 [20]	Poland (Caucasian)	6	482/482	14/182	270	467	227	1007	921	44	101	51	189	203	0.36
Wang 2010 [23]	United States(Mixed)	8	0/102	0/46	38	48	15	124	78	9	26	11	44	48	0.98
Lu 2009 [28]	China(Asian)	7	0/157	0/221	83	56	18	222	92	95	92	34	282	160	0.08
Li 2008 [29]	China(Asian)	6	0/200	0/70	86	82	32	254	146	23	39	8	85	55	0.10
Chen 2008 [30]	China(Asian)	5	36/46	6/76	30	31	21	91	73	37	30	15	104	60	**0.03**
Johansson 2005 [31]	Sweden(Caucasian)	9	180/490	40/220	208	332	130	748	592	83	132	45	298	222	0.90
Lee 2004 [19]	Korean(Asian)	7	0/268	0/137	114	110	44	338	198	46	76	15	106	168	0.05

a The quality score was determinded by using the Newcastle-Ottawa quality assessment scale.

*ESR1*: estrogen receptor 1 gene; M: Male; F: Female; HWE: Hardy-Weinberg equilibrium.

**Table 3:** Characteristics and *ESR1 Xba*I polymorphism genotype distributions in studies included in the meta-analysis.

Author, year	Ethnicity	Quality score^a^	Gender (M/F)		Control					Case					*P*_HWE_
			Control	Case	AA	AG	GG	A	G	AA	AG	GG	A	G	
Wang 2010 [23]	United States(Mixed)	8	0/102	0/46	48	44	9	140	62	14	24	8	52	40	0.81
Lu 2009 [28]	China(Asian)	7	0/157	0/221	112	38	7	262	52	138	73	10	349	93	0.12
Li 2008 [29]	China(Asian)	6	0/200	0/70	144	46	10	334	66	46	19	5	111	29	**0.02**
Chen 2008 [30]	China(Asian)	5	36/46	6/76	45	29	8	119	45	48	31	3	127	37	0.31
Johansson 2005 [31]	Sweden(Caucasian)	9	180/490	40/220	332	281	57	945	395	145	94	21	384	136	0.82
Lee 2004 [19]	Korean(Asian)	7	0/268	0/137	192	62	14	446	90	89	38	10	216	58	**<0.01**

a The quality score was determinded by using the Newcastle-Ottawa quality assessment scale.

*ESR1*: estrogen receptor 1 gene; M: Male; F: Female; HWE: Hardy-Weinberg equilibrium.

### Quantitative synthesis

**Figure 2:**
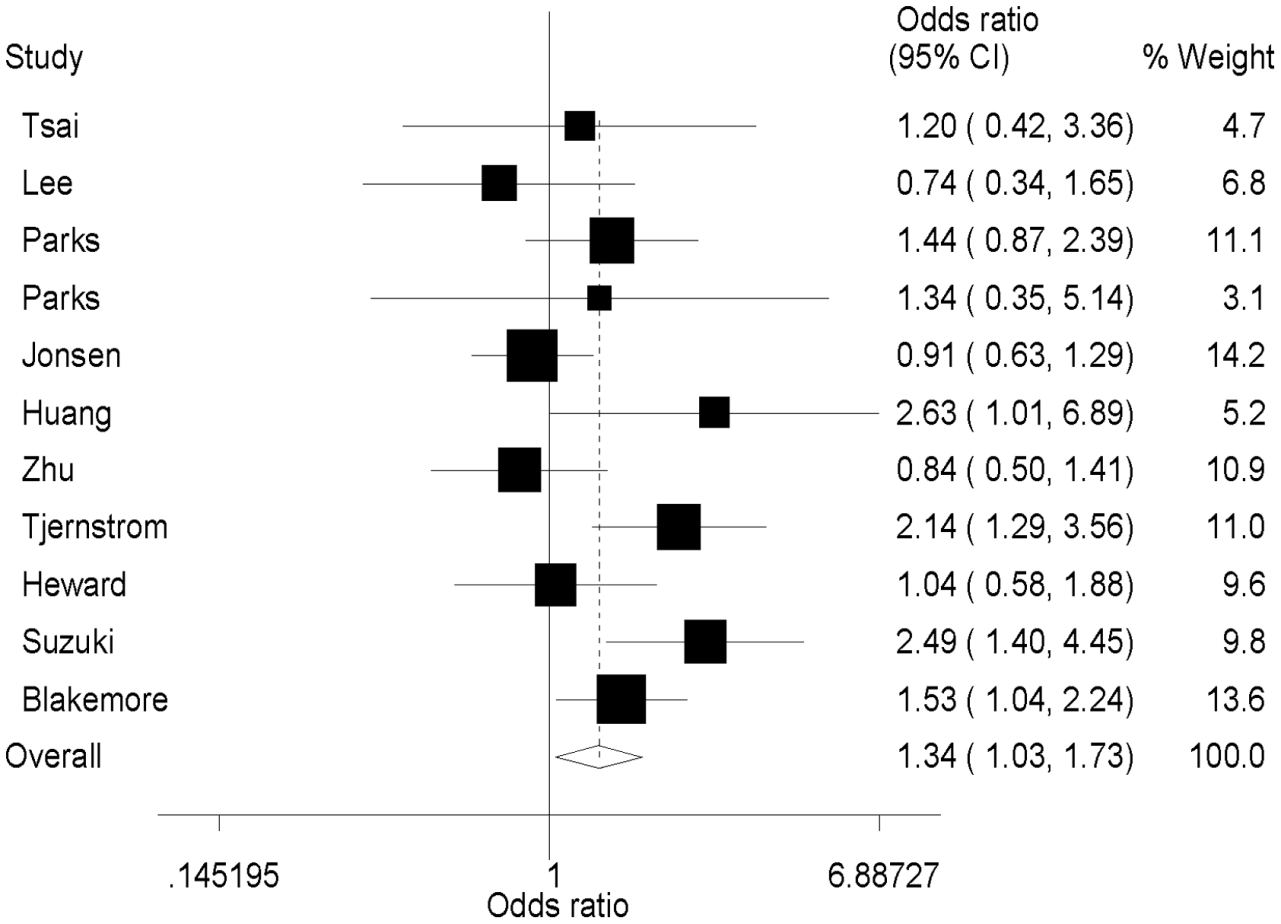
Forest plot of the association between SLE susceptibility and *IL1-RN* VNTR polymorphism (2 versus L).

Eleven studies including 1171 SLE patients and 1834 controls assessed the relationship between *IL1-RN* VNTR polymorphisms and SLE susceptibility. *IL1-RN* VNTR polymorphism showed no significant association with SLE susceptibility in a dominant model (22/2L vs. LL: OR = 1.11, 95% CI = 0.87−1.40, *P* = 0.40), recessive model (22 vs. LL/2L: OR = 1.32, 95% CI = 0.88−1.97, *P* = 0.17) or additive model (22 vs. LL: OR = 1.32, 95% CI = 0.88−1.98, *P* = 0.19, Table 4). However, a significant association was observed in an allelic contrast model (2 vs. L: OR = 1.34, 95% CI = 1.03–1.73, *P* = 0.03, Figure 2). After excluding the studies in which the genotype distributions in the control groups deviated from HWE, the *IL1-RN* VNTR polymorphism showed no significant association with SLE susceptibility in all genetic models (Table S1). We performed subgroup analyses in Asian and Caucasian populations and found no significant differences in either ethnic subgroup (Table S1).

**Table 4:** Main results of meta-analysis of the association of *IL1-RN* VNTR, *ESR1 Pvu*II and *ESR1 Xba*I polymorphisms with SLE susceptibility.

Gene and Genetic models	Number of study	*P*_heterogeneity_	I^2^(%)	Type of effect model	ORs (95% CI)	*P*
*IL1-RN VNTR*						
Dominant (22/2L vs. LL)	8	0.19	29.3	Fixed	1.11 (0.87–1.40)	0.40
Recessive (22 vs. LL/2L)	7	0.50	0	Fixed	1.32 (0.88–1.97)	0.17
Additive (22 vs. LL)	7	0.46	0	Fixed	1.32 (0.88–1.98)	0.19
Allelic contrast (2 vs. L)	11	**0.02**	51.4	Random	**1.34 (1.03**–**1.73)**	**0.03**
*ESR1 Pvu*II						
Dominant (CC/CT vs. TT)	7	0.10	43.3	Fixed	**1.25 (1.06**–**1.47)**	**0.01**
Recessive (CC vs. TT/CT)	7	0.22	26.8	Fixed	0.96 (0.79–1.17)	0.71
Additive (CC vs. TT)	7	0.11	42.5	Fixed	1.10 (0.88–1.38)	0.41
Allelic contrast (C vs. T)	7	**0.00**	85.5	Random	1.28 (0.95–1.74)	0.11
*ESR1 Xba*I						
Dominant(GG/GA vs. AA)	6	**0.03**	58.8	Random	1.19 (0.88–1.62)	0.27
Recessive(GG vs. AA/AG)	6	0.39	6.1	Fixed	1.08 (0.77–1.51)	0.67
Additive(GG vs. AA)	6	0.17	35.1	Fixed	1.09 (0.77–1.54)	0.64
Allelic contrast(G vs. A)	6	**0.03**	60.4	Random	1.15 (0.89–1.49)	0.27

*IL1-RN*: Interleukin-1 receptor antagonist gene; VNTR: variable number of tandem repeats; *ESR1*: estrogen receptor 1 gene; OR: odds ratio; CI: confidence interval.

**Figure 3:**
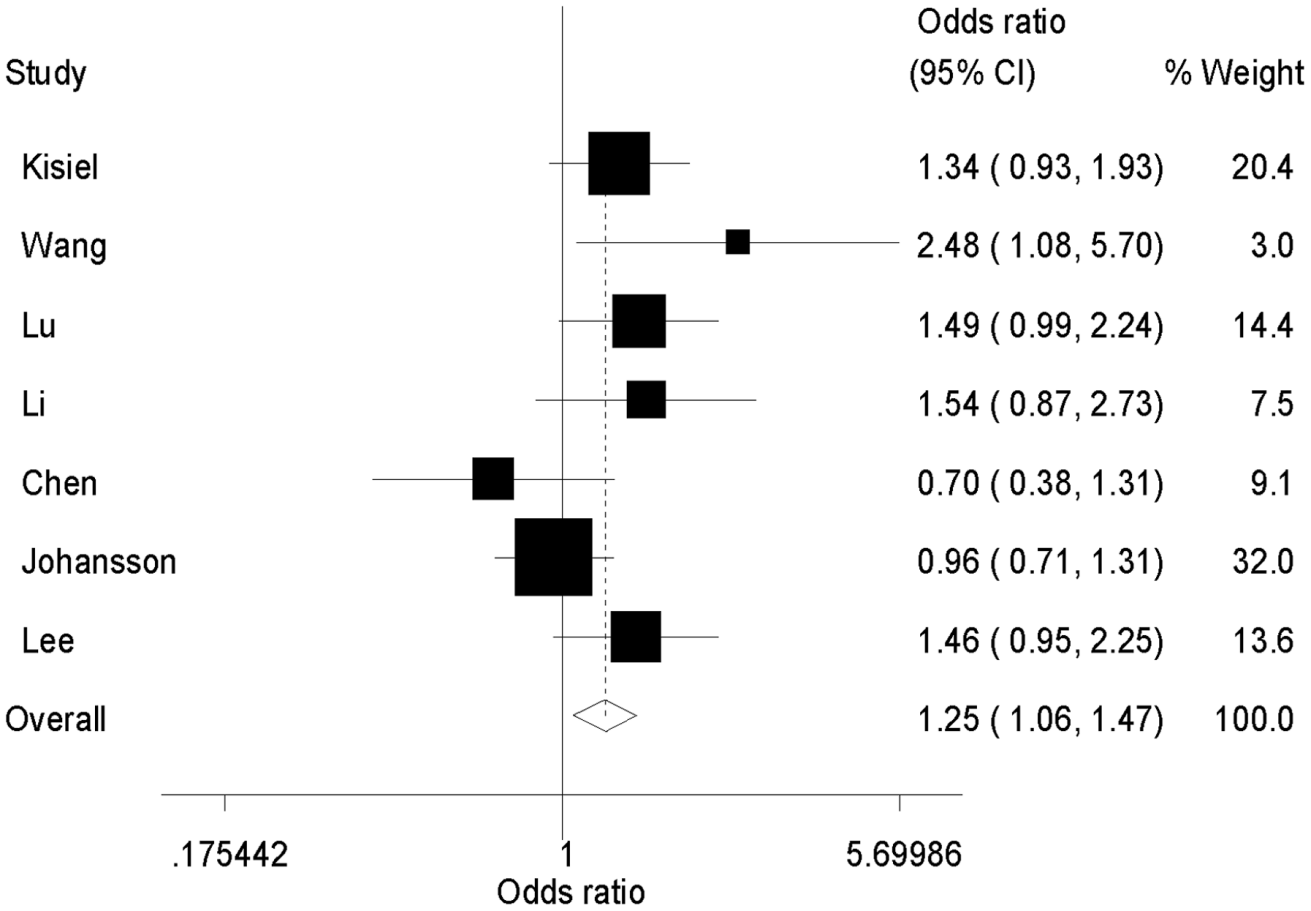
Forest plot of the association between SLE susceptibility and *ESR1 Pvu*II polymorphism (CC/CT versus TT).

Seven studies compared the *ESR1 Pvu*II polymorphism in SLE patients and controls. Individuals carrying variant genotypes had an increased risk of SLE in the dominant model (CC/CT vs. TT: OR = 1.25, 95% CI = 1.06–1.47, *P* = 0.01, Figure 3) but not in other genetic models (CC vs. TT/CT: OR = 0.96, 95% CI = 0.79–1.17, *P* = 0.71; CC vs. TT: OR = 1.10, 95% CI = 0.88–1.38, *P* = 0.41; C vs. T: OR = 1.28, 95% CI = 0.95–1.74, *P* = 0.11, Table 4). After excluding the study in which the genotype distribution in the control group deviated from the HWE, *ESR1 Pvu*II polymorphism was significantly associated with SLE susceptibility in both dominant and allelic contrast models (Table S2). When grouped by ethnicity, a significant association was still observed in the dominant model in the Asian group (CC/CT vs. TT: OR = 1.33, 95% CI = 1.04–1.69, *P* = 0.02, Table S2) but not in the Caucasian group (CC/CT vs. TT: OR = 1.11, 95% CI = 0.88–1.40, *P* = 0.38, Table S2).

**Figure 4:**
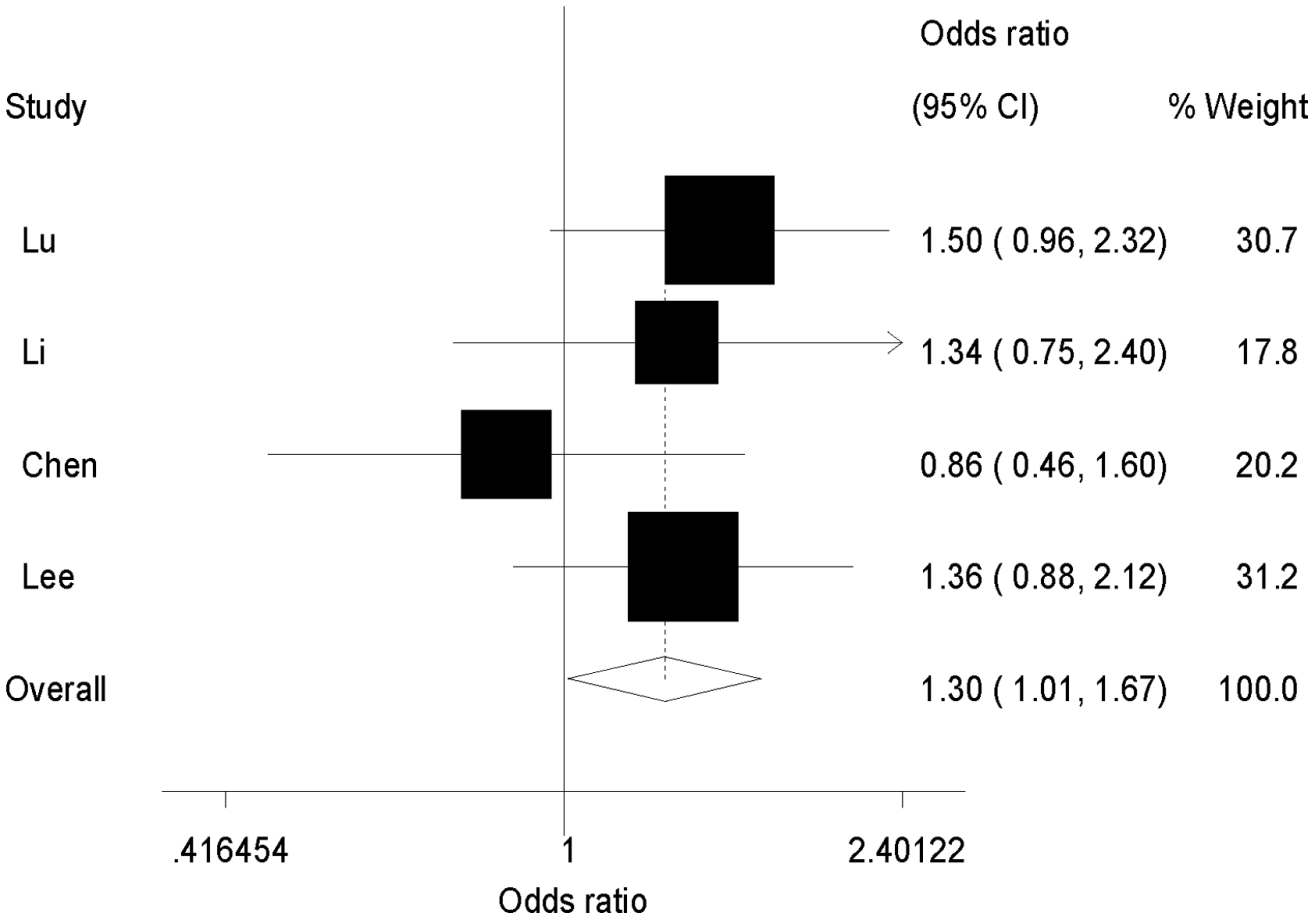
Forest plot of the association between SLE susceptibility and *ESR1 Xba*I polymorphism in Asian descent (GG/GA versus AA).

Six studies investigated the association between *ESR1 Xba*I polymorphism and SLE susceptibility. No significant relationships were identified for any of the genetic models in the whole study set (GG/GA vs. AA: OR = 1.19, 95% CI = 0.88–1.62, *P* = 0.27; GG vs. AA/GA: OR = 1.08, 95% CI = 0.77–1.51, *P* = 0.67; GG vs. AA: OR = 1.09, 95% CI = 0.77–1.54, *P* = 0.64; G vs. A: OR = 1.15, 95% CI = 0.89–1.49, *P* = 0.27, Table 4). Exclusion of the two studies in which the genotype distributions in the control groups deviated from the HWE had no significant effect on the results (Table S3). However, stratified analysis by ethnicity demonstrated a marginally significant association in the dominant model (GG/GA vs. AA: OR = 1.30, 95% CI = 1.01–1.67, *P* = 0.04, Figure 4, Table S3) in individuals of Asian descent.

### Tests of heterogeneity

For *IL1-RN* VNTR, *ESR1 Pvu*II, and *ESR1 Xba*I, heterogeneity between studies was observed in the overall analysis of the allelic contrast model (*P*_heterogeneity_ = 0.02, 0.00, 0.03, respectively, Table 4). In addition, heterogeneity was also found for *ESR1 Xba*I in the dominant model (*P*_heterogeneity_ = 0.03). Ethnicity was assessed as a potential source of heterogeneity. Ethnicity (χ^2^ = 11.49, df = 2, *P* = 0.003) contributed to the heterogeneity for *ESR1 Pvu*II. Ethnicity also contributed to the heterogeneity for the dominant model (χ^2^ = 10.00, df = 2, *P* = 0.007) and the allelic contrast model (χ^2^ = 8.83, df = 2, *P* = 0.010) for *ESR1 Xba*I.

### Sensitivity analysis

Sensitivity analysis revealed that heterogeneity decreased after some studies were removed: Jonsen *et al.* 2004 [21] for *IL1-RN* VNTR (2 vs. L: *P*_heterogeneity_ = 0.08, I^2^ = 41.8%); Johansson *et al.* 2005 [31] for *ESR1 Xba*I (G vs. A: *P*_heterogeneity_ = 0.25, I^2^ = 26.4%); and Johansson *et al.* 2005 [31] for *ESR1 Xba*I (GG/GA vs. AA: *P*_heterogeneity_ = 0.48, I^2^ = 0.0%). The results of the association between *ESR1 Pvu*II and SLE susceptibility were not substantially altered.

### Publication bias

**Figure 5:**
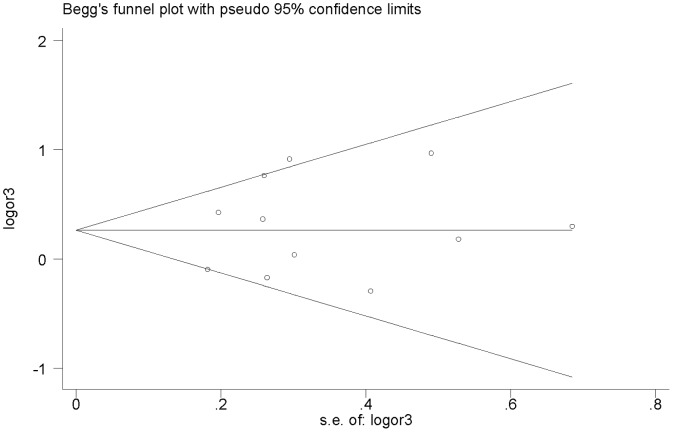
Begg’s funnel plot for publication bias test. *IL1-RN* VNTR: 2 versus L.

No publication bias was detected among studies regarding the association between the *IL1-RN* VNTR polymorphism and SLE (*P* = 0.83 for 2 vs. L, Figure 5). Similarly, the results of Egger’s and Begg’s tests showed no publication bias for the *ESR1 Pvu*II or *ESR1 Xba*I polymorphisms in all models.

## Discussion

Studies of gene polymorphisms potentially related to SLE have recently attracted growing attention. In the present study, we performed a meta-analysis of the associations between *IL1-RN* VNTR, *ESR1 Pvu*II, and *ESR1 Xba*I polymorphisms and SLE susceptibility. The analysis indicated an association between the 2 allele of the VNTR polymorphism in intron 2 of *IL1-RN* and increased SLE susceptibility (2 vs. L: OR = 1.34, 95% CI = 1.03–1.73, *P* = 0.03). There was also an association between *ESR1 Pvu*II and SLE in the dominant model (CC/CT vs. TT: OR = 1.25, 95% CI = 1.06–1.47, *P* = 0.01), which was pronounced among Asian individuals (CC/CT vs. TT: OR = 1.33, 95% CI = 1.04–1.69, *P* = 0.02). There was no significant association between the *ESR1 Xba*I polymorphism and SLE susceptibility in the overall analysis, but the GG/GA genotype was associated with SLE susceptibility in Asians (GG/GA vs. AA: OR = 1.30, 95% CI = 1.01–1.67, *P* = 0.04).

IL1 is a potent pro-inflammatory cytokine in acute and chronic inflammation in SLE [37]. IL1RA is a natural antagonist of IL1 and its anti-inflammatory activity is mediated through several different pathways [38] and investigations found decreased production of IL1RA in active SLE [39]. The *IL1-RN* VNTR 2 allele was associated with increased production of IL1β *in vitro* [40], [41] and the concentration of IL1RA was shown to be correlated with IL1β [42]. Also, this meta-analysis identified carriage of the 2 allele as a risk factor for SLE susceptibility (2 vs. L: OR = 1.34, 95% CI = 1.03–1.73, *P* = 0.03). In support of this, the *IL1-RN* VNTR contains three potential protein-binding sites: an acute phase response element, an interferon α and an interferon β silencer B [43]. The 2 allele of *IL1-RN* VNTR only has 2 repeats. This could affect mRNA length and subsequent protein processing and stability [44], which could in turn affect the production of IL1RA.

Our study also showed that the *ESR1 Pvu*II CC/CT and *ESR1 Xba*I GG/GA genotypes could increase susceptibility to SLE. Estrogen can affect both innate and adaptive immune responses in mice [45] and SLE patients [46] through different pathways [47]–[49], and estrogen receptors are expressed in most immunocompetent cells [50]. Some researchers determined that IL1β levels were higher during the luteal period compared with the follicular period of the female reproductive cycle, which was consistent with the results of *in vivo* [51]–[53] and *in vitro* tests [18]. The polymorphisms *Pvu*II and *Xba*I are located in intron 1 of *ESR1* but are still able to affect the gene, and thus affect estrogen concentrations. The C allele of *Pvu*II can produce a binding site for the B-myb transcription factor, which could enhance the ability to up-regulate downstream receptor structures compared with the T allele [54]. In the present study, SLE susceptibility was associated with the *ESR1 Pvu*II C allele but not with *Xba*I in overall analysis. However, we could not rule out the possibility of an association between the *ESR1 Xba*I polymorphism and SLE susceptibility because *Pvu*II and *Xba*I are tightly linked [55] and it is difficult to identify which one has a role to play.

Given its multifactorial nature, it is likely that the pathogenesis of SLE may be modulated by age, gender, ethnicity, environmental factors and other variables. We therefore carried out subgroup analysis based on ethnicity. Associations between SLE susceptibility and the *ESR1 Pvu*II C allele and the *ESR1 Xba*I G allele were found in individuals of Asian descent. This may be attributable to genetic heterogeneity among different populations. Consistent with this, ethnicity contributed to the heterogeneity for *ESR1 Pvu*II and *ESR1 Xba*I. Moreover, sensitivity analysis revealed that the heterogeneity was reduced by removing Johansson *et al*.’s [31] study, which was the only study in the meta-analysis of the association between *ESR1 Xba*I polymorphism and SLE susceptibility that was based on Caucasians. It is also possible that differences in lifestyle and environmental factors between different populations may interact with genes to affect the pathogenesis of SLE.

This meta-analysis had some inevitable limitations. First, three studies on the *IL1-RN* VNTR polymorphism did not provide genotype data, and the data used to analyze the various genetic models were thus not completely consistent. This may lead to misinterpretation of the association between the *IL1-RN* VNTR polymorphism and SLE susceptibility. Second, although all eligible studies were included in our study, the small sample size and low statistical power (Table S4, S5 and S6) associated with the low incidence of SLE means that there is a possibility of false negative results. We expect more participants being tested in the future to draw a more reliable conclusion. Third, deviation of genotype distributions from HWE in the control populations in some studies may reflect genotyping errors or control selection bias. The results relating to *IL1-RN* VNTR and *ESR1 Pvu*II changed when these studies were excluded, suggesting that these results should be interpreted with caution. Fourth, as mentioned above, various factors affect the pathology of SLE; the lack of individual data meant that we only pooled the data based on unadjusted information. Finally, the quality scores were not high for some studies and these studies may have distorted the results.

In conclusion, this meta-analysis indicated that the *IL1-RN* VNTR 2 allele and the *ESR1 Pvu*II CC/CT and *ESR1 Xba*I GG/GA genotypes may increase the susceptibility to SLE, especially in individuals of Asian descent. However, this conclusion should be interpreted cautiously because of the low statistical power and considerable heterogeneity. Also, further functional studies are needed to investigate the functions of these alleles. Well-designed, large studies in different ethnic groups and with more detailed information on age, sex, and age of onset of the disease are needed to validate our results. Studies of gene-environment interactions in relation to *IL1-RN*-*ESR1* should also be performed to confirm our preliminary findings.

## Supporting Information

Table S1 **Meta analysis of the association of** ***IL1-RN VNTR*** **polymorphism with SLE susceptibility.**
(XLS)

Table S2 **Meta analysis of the association of** ***ESR1 Pvu*** **II polymorphism with SLE susceptibility.**
(XLS)

Table S3 **Meta analysis of the association of** ***ESR1 Xba*** **I polymorphism with SLE susceptibility.**
(XLS)

Table S4 **The statistical power of** ***IL1-RN VNTR*** **2 allele in studied included in the meta-analysis.**
(XLS)

Table S5 **The statistical power of** ***ESR1 Pvu*** **II CC/CT genotypes in studied included in the meta-analysis.**
(XLS)

Table S6 **The statistical power of** ***ESR1 Xba*** **I GG/GA genotypes in studied included in the meta-analysis.**
(XLS)
